# Sub-genotype phylogeny of the non-G, non-P genes of genotype 2 *Rotavirus A* strains

**DOI:** 10.1371/journal.pone.0217422

**Published:** 2019-05-31

**Authors:** Chantal Ama Agbemabiese, Toyoko Nakagomi, Susan Afua Damanka, Francis Ekow Dennis, Belinda Larteley Lartey, George Enyimah Armah, Osamu Nakagomi

**Affiliations:** 1 Department of Electron Microscopy and Histopathology, Noguchi Memorial Institute for Medical Research, College of Health Sciences, University of Ghana, Legon, Accra, Ghana; 2 Department of Molecular Epidemiology, Graduate School of Biomedical Sciences, Nagasaki University, Nagasaki, Japan; Universidade Nova de Lisboa Instituto de Higiene e Medicina Tropical, PORTUGAL

## Abstract

Recent increase in the detection of unusual G1P[8], G3P[8], G8P[8], and G9P[4] *Rotavirus A* (*RVA*) strains bearing the DS-1-like constellation of the non-G, non-P genes (hereafter referred to as the genotype 2 backbone) requires better understanding of their evolutionary relationship. However, within a genotype, there is lack of a consensus lineage designation framework and a set of common sequences that can serve as references. Phylogenetic analyses were carried out on over 8,500 RVA genotype 2 genes systematically retrieved from the rotavirus database within the NCBI Virus Variation Resource. In line with previous designations, using pairwise comparison of cogent nucleotide sequences and stringent bootstrap support, reference lineages were defined. This study proposes a lineage framework and provides a dataset ranging from 34 to 145 sequences for each genotype 2 gene for orderly lineage designation of global genotype 2 genes of RVAs detected in human and animals. The framework identified five to 31 lineages depending on the gene. The least number of lineages (five to seven) were observed in genotypes A2 (NSP1), T2 (NSP3) and H2 (NSP5) which are limited to human RVA whereas the most number of lineages (31) was observed in genotype E2 (NSP4). Sharing of the same lineage constellations of the genotype 2 backbone genes between recently-emerging, unusual G1P[8], G3P[8], G8P[8] and G9P[4] reassortants and many contemporary G2P[4] strains provided strong support to the hypothesis that unusual genotype 2 strains originated primarily from reassortment events in the recent past involving contemporary G2P[4] strains as one parent and ordinary genotype 1 strains or animal RVA strains as the other. The lineage framework with selected reference sequences will help researchers to identify the lineage to which a given genotype 2 strain belongs, and trace the evolutionary history of common and unusual genotype 2 strains in circulation.

## Introduction

*Rotaviruses A* (RVA) within the genus *Rotavirus* of the *Reoviridae* family, are a major cause of severe acute gastroenteritis in children and the young of various mammals and birds. RVAs possess a triple-layered capsid that contains a genome of 11 segments of double-stranded RNA encoding six structural viral proteins (VP1-VP4, VP6, VP7) and five to six non-structural proteins (NSP1-NSP5/NSP6) [1].

RVA strains are classified into G and P genotypes defined by the nucleotide sequence identity cut-off values for the VP7 and VP4 genes, respectively [1]. A complete genome-based classification system differentiates two major and one minor genotype constellations of human RVA strains: they are Wa-like or genotype 1 constellation (G1/G3/G4/G9/G12-P[8]-I1-R1-C1-M1-A1-N1-T1-E1-H1), DS-1-like or genotype 2 constellation (G2-P[4]-I2-R2-C2-M2-A2-N2-T2-E2-H2) and AU-1-like or genotype 3 constellation (G3-P[9]-I3-R3-C3-M3-A3-N3-T3-E3-H3) [2–4].

The RVA triple layered particle consists of an inner core (VP2), a middle layer (VP6) and an outer layer (VP7) with spikes of the VP4 protruding from the outer capsid. Located within the VP2 core are the VP1 and VP3 which are the viral RNA dependent RNA polymerase and methyl transferase (capping enzyme) respectively. The non-structural proteins NSP1-NSP5/NSP6 play diverse roles during the complex virus replication cycle that is orchestrated by an interplay between the rotavirus structural and non-structural proteins. These inner capsid proteins (VP1, VP2, VP3, and VP6) and the non-structural protein genes (hereafter referred to as the backbone genes) of the genotype 2 strains have recently attracted renewed attention. Firstly, on the African continent, it is common to find G2P[6], G3P[6], G6P[6], G8P[4], G8P[6], G8P[8], and G9P[6] RVA strains possessing the genotype 2 backbone genes in human RVA strains [5–9]. Secondly, the genotype 2 backbone has been found in association with P[8] strains that usually carry genotype 1 backbone such as G1P[8] [10–14], G3P[8] [15–24], and G8P[8] [25–28]. In addition, G1P[4], G3P[4], G9P[4] and G12P[6] strains have been reported to possess the genotype 2 backbone genes [29–37].

However, the evolutionary relationships among these many, recently emerging genotype 2 strains possessing different G and P genotypes as well as their relationships to historical and contemporary G2P[4] strains are not very clear due to the lack of a common framework in the designation of lineages below the level of genotypes. Difficulty in understanding the similarity between two strains will increase when the cognate genes of these strains are placed in the phylogenetic trees in which different reference sequences are used as references for the lineages. There were at least three groups of investigators who proposed a framework for the classification of the genotype 2 backbone genes by successively revising and updating their predecessors’ achievement [31, 38, 39]. Giammanco et al. [38] described the evolution of Italian G2P[4] strains by defining the lineages considering data of the existing literature and substantiated the lineages by sequence comparison and neighbour joining phylogenetic analysis. In describing the evolution of Japanese G2P[4] strains over a 32 year period, Doan et al. [39] provided a comprehensive framework for the genotype 2 genes by defining the lineages based on a consistent clustering pattern of genes across the genome of each G2P[4] strain. Subsequent studies adopted the lineage classification of Doan et al. (2015) to successfully describe the evolutionary pattern of local G2P[4] strains obtained from Vietnam, Ghana, and Bangladesh within a global context [40–42]. This lineage classification system was also shown to be useful in understanding the precise relationship between globally emerging DS-1-like G1P[8] strains detected in Vietnam [11], equine-like G3P[8] strains detected in Japan and Brazil [17, 24] as well as emerging G9P[4] strains in eastern India [33]. Pradhan et al. [31], improving the consistency in the bootstrap support and redefining divergence limits between lineages, proposed another lineage classification system where they included non-G2P[4] human RVA strains bearing the genotype 2 backbone genes, and characterized the genotype 2 backbone genes of G9P[4] strains from western India.

However, Pradhan et al. [31] did not include the genotype 2 genes of animal RVAs. The inclusion of genotype 2 genes of animal RVAs in the phylogenetic framework is indispensable because, firstly, human genotype 2 strains share common ancestral origins with bovine rotaviruses [2]; and secondly, non-G2P[4] strains with the genotype 2 backbone sometimes possess a few genotype 2 genes of animal RVA origin [7, 9, 43, 44]. While the need for an all-inclusive framework for classification of genotype 2 genes is clear, a practical problem is the selection of appropriate reference sequences for appropriate assignment of genomic inter-relationships at the sub-genotype/lineage level. Due to lack of convention in describing close phylogenetic relationship among sequences, the tendency to refer to the closest possible strain available in the GenBank database when carrying out BLAST analyses has become the norm.

The aim of this study is therefore to propose lineage designations within genotype 2 genes by using a set of reference strains that can be shared by rotavirus researchers, and to provide a phylogenetic basis on which the emergence and evolution of unusual reassortant strains can be interpreted at the lineage level, i.e. one step more precisely than at the genotype level.

## Materials and methods

### The source of sequence data used in this study

**Fig 1:**
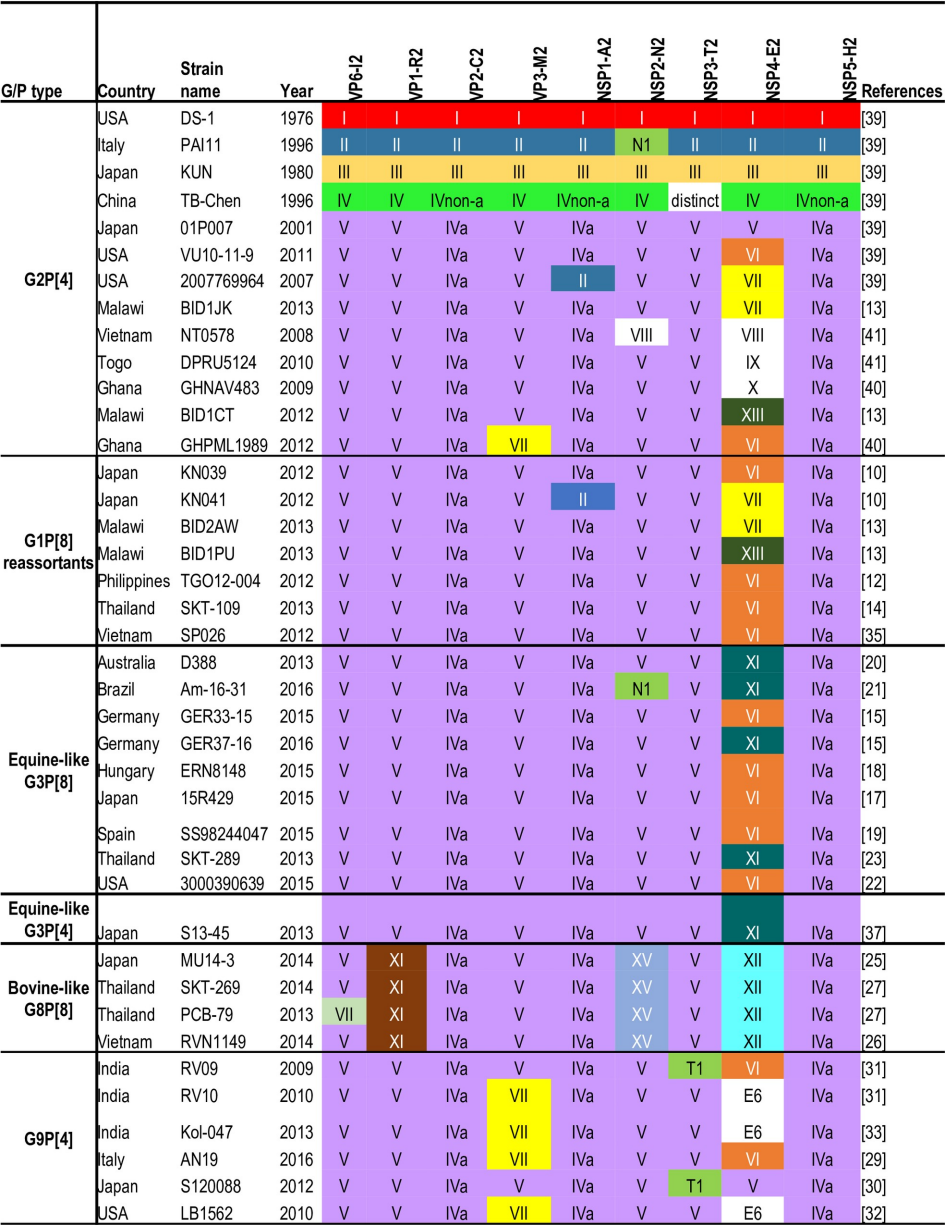
Lineage constellation of representative G2P[4] and representative genotype 2 reassortant strains. Lineage colours for I-V, VII and VII (for VP3 and NSP4) were adapted from Doan et al.’s [39] G2P[4] lineage designation.

Nucleotide sequence data for the nine backbone (non-G, non-P) genes of genotype 2 strains, namely: VP1-R2, VP2-C2, VP3-M2, VP6-I2, NSP1-A2, NSP2-N2, NSP3-T2, NSP4-E2, and NSP5-H2 were retrieved from the rotavirus database available on the NCBI Virus Variation Resource (https://www.ncbi.nlm.nih.gov/genomes/VirusVariation/Database/nph-select.cgi?taxid=28875). The NCBI Viral Genomes Resource is a reference resource designed to bring order in this era of viral genome sequence explosion as well as to improve usability of viral sequence data [45, 46]. Also included in this study were recently emerging, unusual reassortant strains bearing the genotype 2 backbone. These include G1P[8], G3P[8], G8P[8], and G9P[4] strains [15–24, 29–31, 33]. Using the established lineage framework in this study, the lineage constellations of the backbone genes of the unusual reassortants strains were succinctly described (Fig 1).

### The search criteria and the strategy for selecting sequences for the final dataset

The rotavirus database of the Virus Variation Resource was searched using the filters specified as follows: the RVA species, host (any), region/country (any), segment (specified for the gene of interest e.g. VP1), isolation source (any). Under the additional filters section, the gene of interest was selected, and the corresponding genotype 2 was specified. At the start of systematic decimation steps, sequences shorter than the 50% of the entire length of each of the nine genes were excluded. Then, multiple sequence alignments were generated by using the alignment analysis tool in the Virus Variation Resource, further curated using the MEGA 6.06 software [47] and re-aligned using the online version of Multiple Alignment using Fast Fourier Transform (MAFFT version 7; https://mafft.cbrc.jp/alignment/software/) [48].

Preliminary phylogenetic trees were constructed using the neighbour-joining method with the Kimura-2-parameter model and 1000 bootstrap replicate trials using the MEGA 6 software. Systematic decimation steps were repeated to identify lineages considering the bootstrap values and the p-distances until each lineage was represented by a minimum number of sequences of at least two in the finalised dataset. Where a sequence does not form a lineage with any other sequence, the sequence is labelled as “distinct”. Final phylogenetic trees were constructed by the Maximum Likelihood method using 1000 bootstrap replicate trials. The best-fit nucleotide substitution model for each gene’s dataset was selected based on the lowest Bayesian Information Criterion score [49] upon model testing in MEGA 6 software. The best fit models selected were as follows: Tamura 3-parameter (T92) model with the discrete Gamma distribution (G) and invariant sites (I) was used for the I2-VP6, A2-NSP1, N2-NSP2, T2-NSP3 sequences; the T92 + G model for the H2-NSP5 sequences; the Tamura Nei (TN93) + G + I for the R2-VP1 sequences; and the General Time Reversible (GTR) model with G + I for the C2-VP2, M2-VP3, and E2-NSP4 sequences.

In designating lineages, the already established lineages I–V (VI and VII inclusive for NSP4 and VP3) for human RVA genotype 2 sequences from G2P[4] strains by Doan et al. [39] were maintained. Indeed, these lineages: lineage I (DS-1/1976, CK20001/1977, and 83A001/1983), lineage II (AK26/1982, D205/1989, and PAI11/1996), lineage III (KUN/1980, and 80S001/1980), and lineage IV (e.g. AU605/1986 and TB-Chen/1996; sometimes designated IVnon-a depending on genome segment) contain historical strains; it is therefore logical that they are maintained in the already designated lineages as it will be impractical to classify them together with contemporary strains detected after 2000.

Updated lineage numbers VIII, IX [41], X [40], and XI [11] identified in the E2-NSP4 genotype were also maintained. In designating new lineages in this study, the following expedient criteria were considered. First, sequences considered to belong to the same lineage needed to have diverged from a single ancestral sequence and had to be supported by a high bootstrap value of ≥70%. Second, as much as possible, the maximum nucleotide sequence diversity within each lineage was limited to 8.5%.

## Results

### Host species origin of the RVA genotype 2 genes

**Fig 2:**
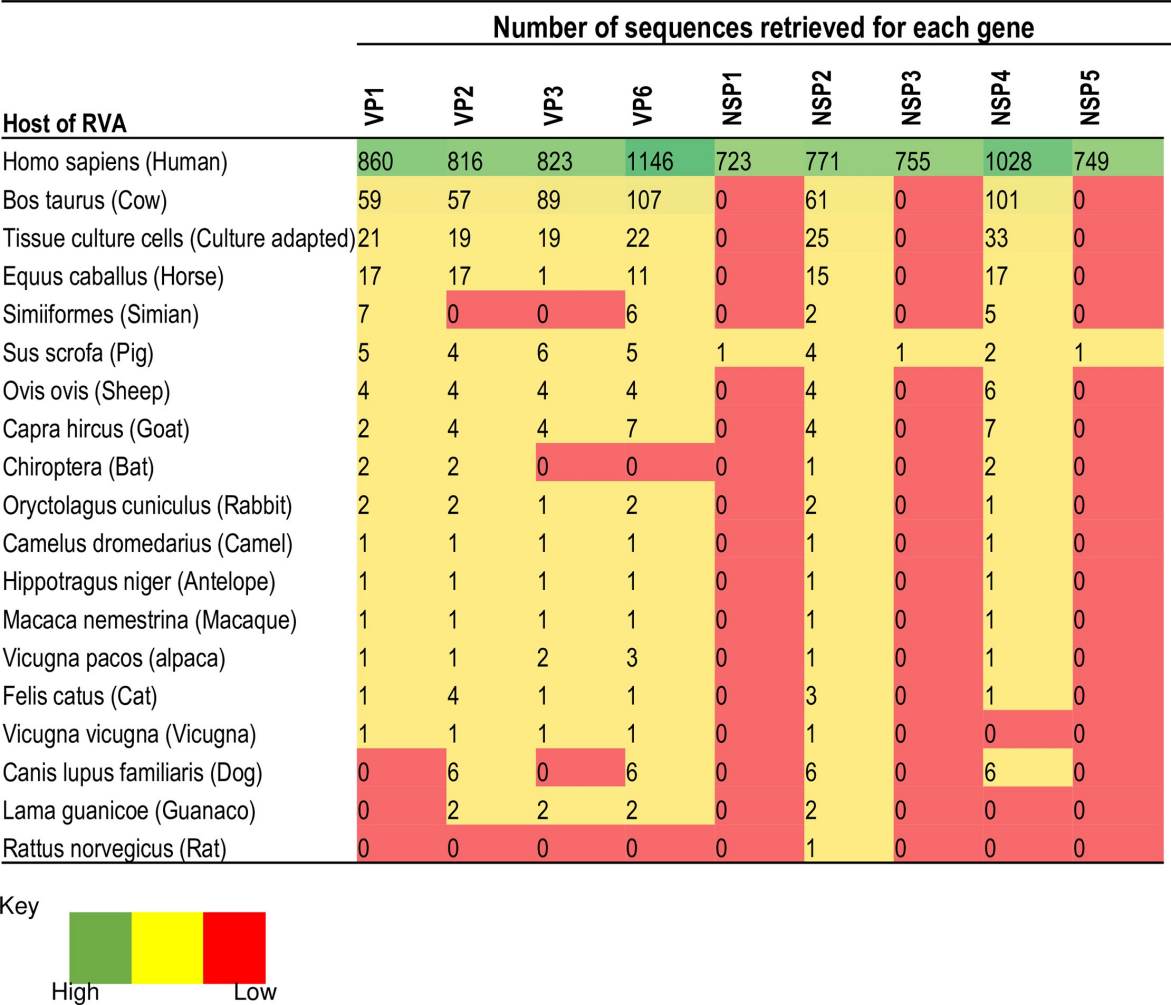
Host distribution/isolation source of genotype 2 genes retrieved from the GenBank using the virus variation rotavirus resource.

The genotype 2 genes were found in RVA of human as well as a diverse range of animal host species origin. Examination of over 8,500 RVA genotype 2 genes retrieved from the NCBI Viral Genomes Resource (Table 1) revealed that six genotypes I2 (VP6), R2 (VP1), C2 (VP2), M2 (VP3), N2 (NSP2), and E2 (NSP4) were detected in a diverse pool of hosts whereas the A2 (NSP1), T2 (NSP3), and H2 (NSP5) genotypes were unique to human RVA strains (Fig 2, S1 Fig). For the structural protein genes, the I2 genotype was detected in 17 host species, R2 16 host species, C2 17 host species, and M2 15 host species. For the non-structural protein genes, the N2 genotype was detected in 19 host species, and E2 16 host species. However, for each of these genes, human and cow RVA strains were the majority, accounting for more than 90% of sequences retrieved from different host species. Less frequent host species thus far reported were equine, ovine, caprine, simian canine and feline RVA strains in the order of overall frequencies.

**Table 1:** Datasets for phylogenetic analysis and average nucleotide sequence divergence within and between lineages of genotype 2 RVA strains.

Gene/Genotype	Starting no. of sequences	No. of reference sequences	Lineages designated	Total no. of lineages	Genetic distance within lineage (%)	Genetic distance between lineages (%)
VP6/I2	1326	64	I—XV	15	0.00–5.25	2.30–15.30
VP1/R2	985	57	I—XIV	14	0.03–6.60	2.40–15.60
VP2/C2	942	65	I—XIV	14	0.00–5.80	2.50–15.70
VP3/M2	956	74	I—XVI	16	0.00–8.50	2.70–18.30
NSP1/A2	724	41	I—V	5	1.30–2.90	3.70–7.80
NSP2/N2	907	78	I—XXII	22	0.00–7.70	3.50–16.70
NSP3/T2	756	43	I—VII	7	0.10–2.30	1.90–9.50
NSP4/E2	1213	145	I—XXXI	30	0.00–7.10	3.80–17.80
NSP5/H2	750	34	I—V	5	0.00–2.70	1.60–7.30

NB: Overlaps in genetic distance values of within and between lineages are due to maintenance of previously designated lineages (I–IV/V).

### Selection of the reference genotype 2 sequences representing each lineage as the final dataset

Out of 724–1,326 sequences per gene of both human and animal rotaviruses (Table 1), retrieved from the NCBI Viral Genomes Resource, short incomplete sequences were removed from the datasets. The resulting datasets for each genome segment were used to construct a neighbour-joining phylogenetic tree, and clustering of sequences was examined to define the lineages by taking into consideration bootstrap values at nodes and the sequence divergence between the sequences within the cluster. Then stepwise decimation was done, by trial and error, with the manual removal of sequences from the dataset until each sub-genotype lineage was represented by a minimal number of sequences (S1 File.) while keeping the topology of the initial clustering pattern intact as much as possible. As summarised in Table 1, the final datasets thus generated contained from 34 (the NSP5 gene) to 124 (the NSP4 gene) sequences which represent 4.5% (the NSP5 gene) to 10.0% (the NSP4 gene) of the starting number of nucleotide sequences retrieved from the database.

### Characteristics of the phylogenetic framework for genotype 2 backbone genes

**Fig 3:**
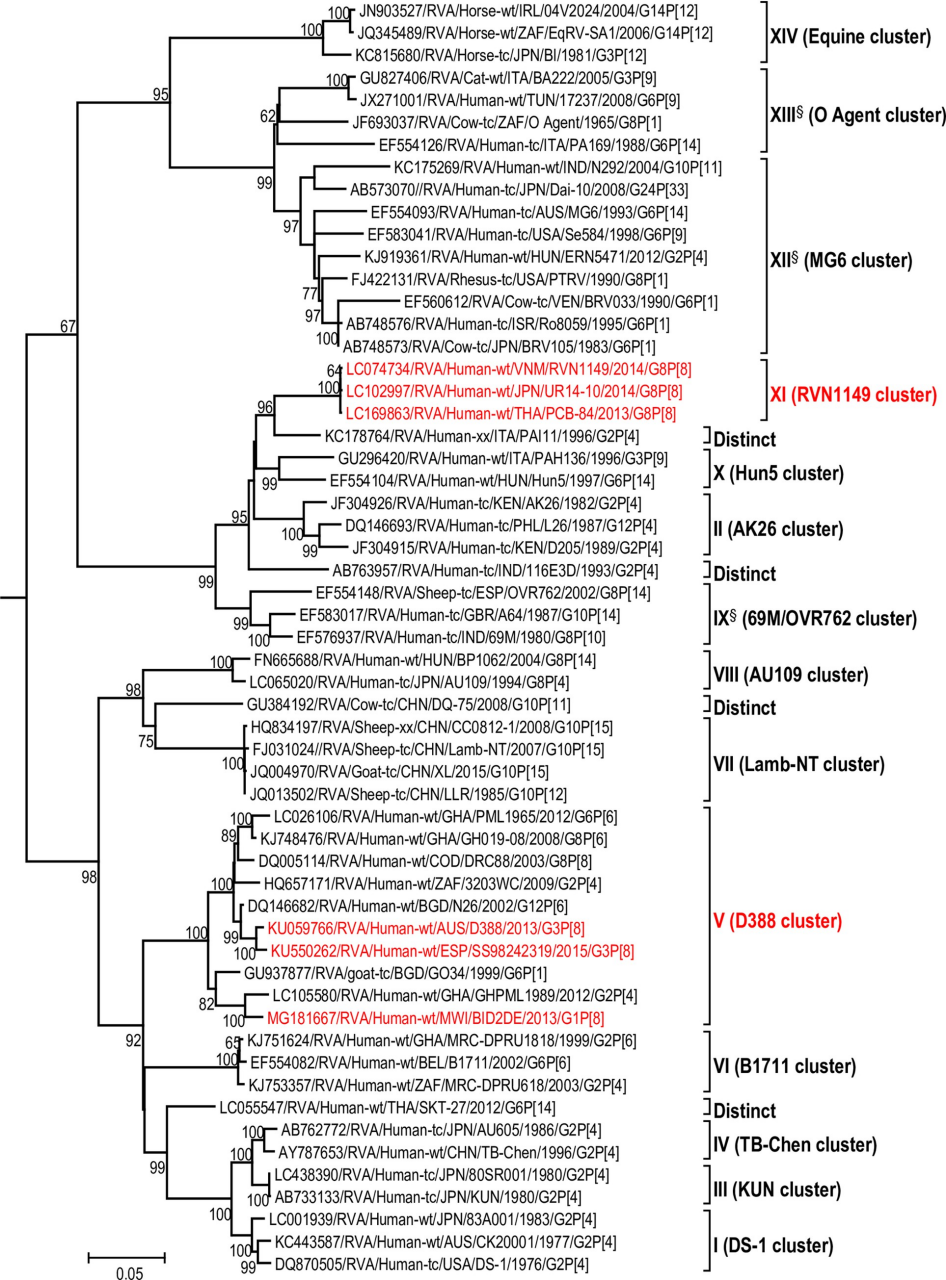
Reference phylogenetic lineage designation for the VP1-R2 genotype of RVA strains of human and animal host species origin. Phylogenetic tree was constructed using the maximum likelihood method with 1000 bootstrap replicate trials. The tree was rooted using the VP1-R1 gene of the genotype 1 prototype strain Wa. “Hybrid lineages” containing a mixture of human and animal RVA sequences are indicated with § in addition to the lineage number. Representative unusual emerging reassortant strains whose lineage constellations were determined in this study using the newly established phylogenetic framework are indicated in red font. Scale bar at the bottom of the tree represents genetic distance expressed as nucleotide substitutions/site.

**Fig 4:**
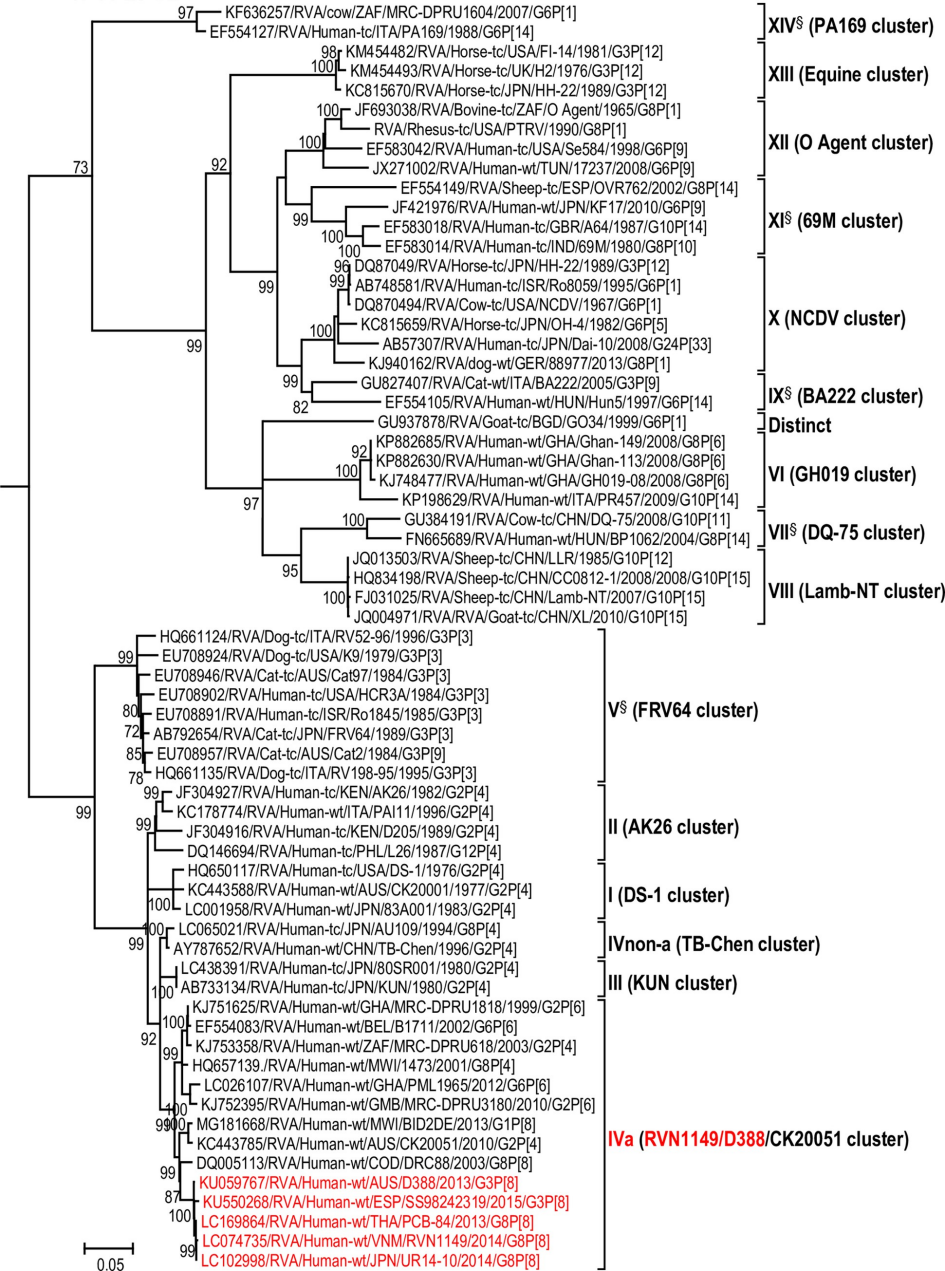
Reference phylogenetic lineage designation for the VP2-C2 genotype of RVA strains of human and animal host species origin. Phylogenetic tree was constructed using the maximum likelihood method with 1000 bootstrap replicate trials. The tree was rooted using the VP2-C1 gene of the genotype 1 prototype strain Wa. “Hybrid lineages” containing a mixture of human and animal RVA sequences are indicated with § in addition to the lineage number. Representative unusual emerging reassortant strains whose lineage constellations were determined in this study using the newly established phylogenetic framework are indicated in red font. Scale bar at the bottom of the tree represents genetic distance expressed as nucleotide substitutions/site.

**Fig 5:**
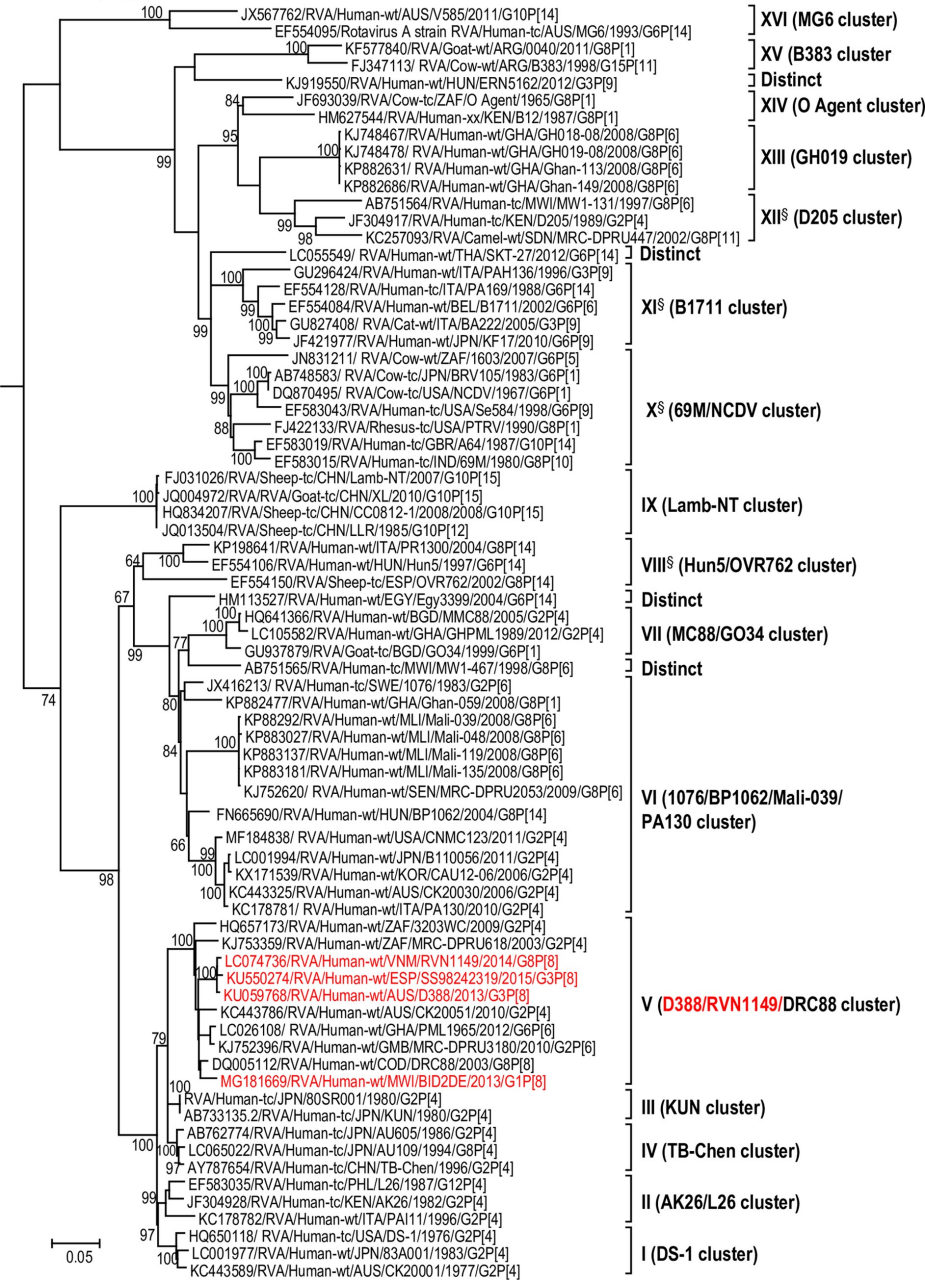
Reference phylogenetic lineage designation for the VP3-M2 genotype of RVA strains of human and animal host species origin. Phylogenetic tree was constructed using the maximum likelihood method with 1000 bootstrap replicate trials. The tree was rooted using the VP3-M1 gene of the genotype 1 prototype strain Wa. “Hybrid lineages” containing a mixture of human and animal RVA sequences are indicated with § in addition to the lineage number. Representative unusual emerging reassortant strains whose lineage constellations were determined in this study using the newly established phylogenetic framework are indicated in red font. Scale bar at the bottom of the tree represents genetic distance expressed as nucleotide substitutions/site.

**Fig 6:**
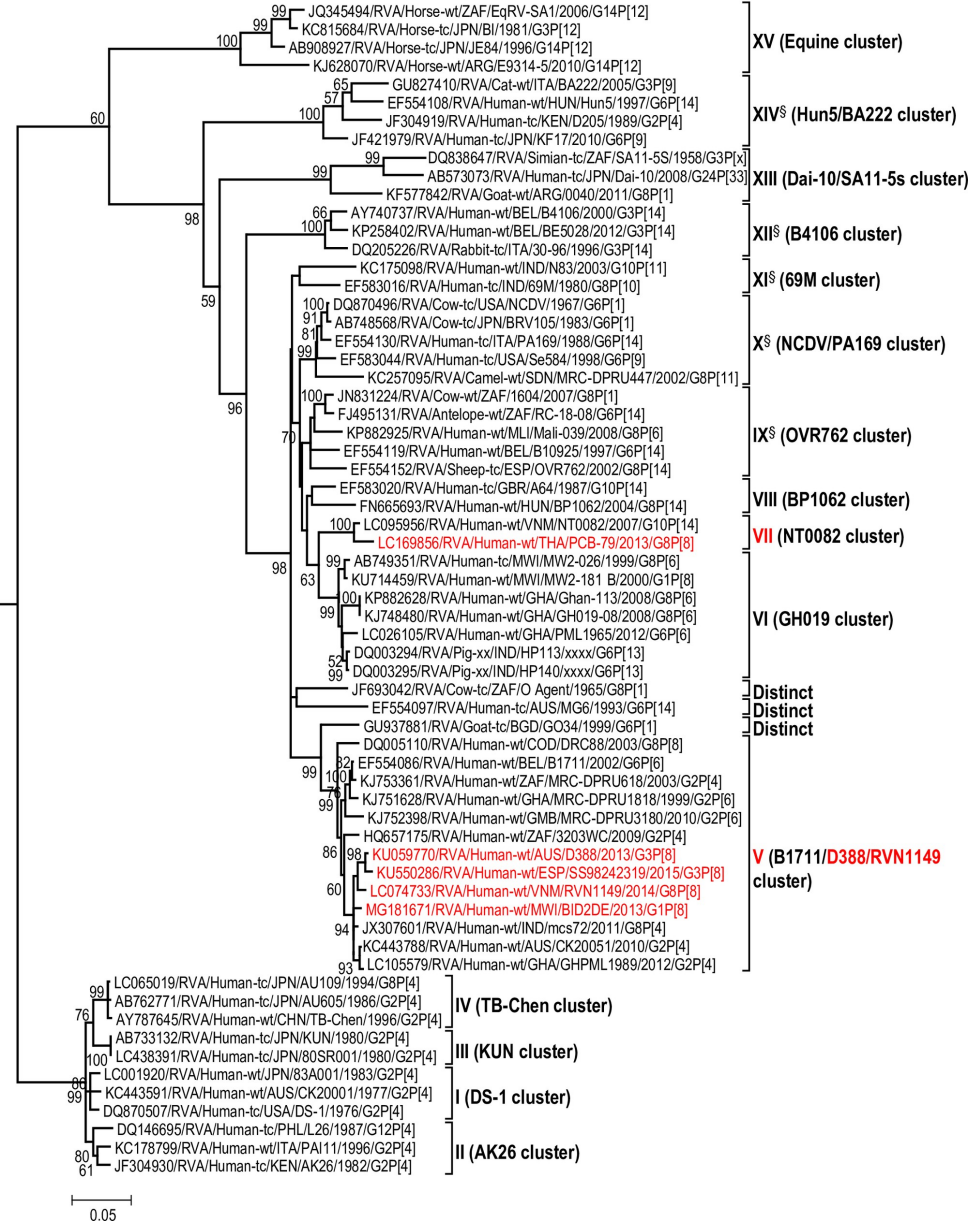
Reference phylogenetic lineage designation for the VP6-I2 genotype of RVA strains of human and animal host species origin. Phylogenetic tree was constructed using the maximum likelihood method with 1000 bootstrap replicate trials. The tree was rooted using the VP6-I1 gene of the genotype 1 prototype strain Wa. “Hybrid lineages” containing a mixture of human and animal RVA sequences are indicated with § in addition to the lineage number. Representative unusual emerging reassortant strains whose lineage constellations were determined in this study using the newly established phylogenetic framework are indicated in red font. Scale bar at the bottom of the tree represents genetic distance expressed as nucleotide substitutions/site.

**Fig 7:**
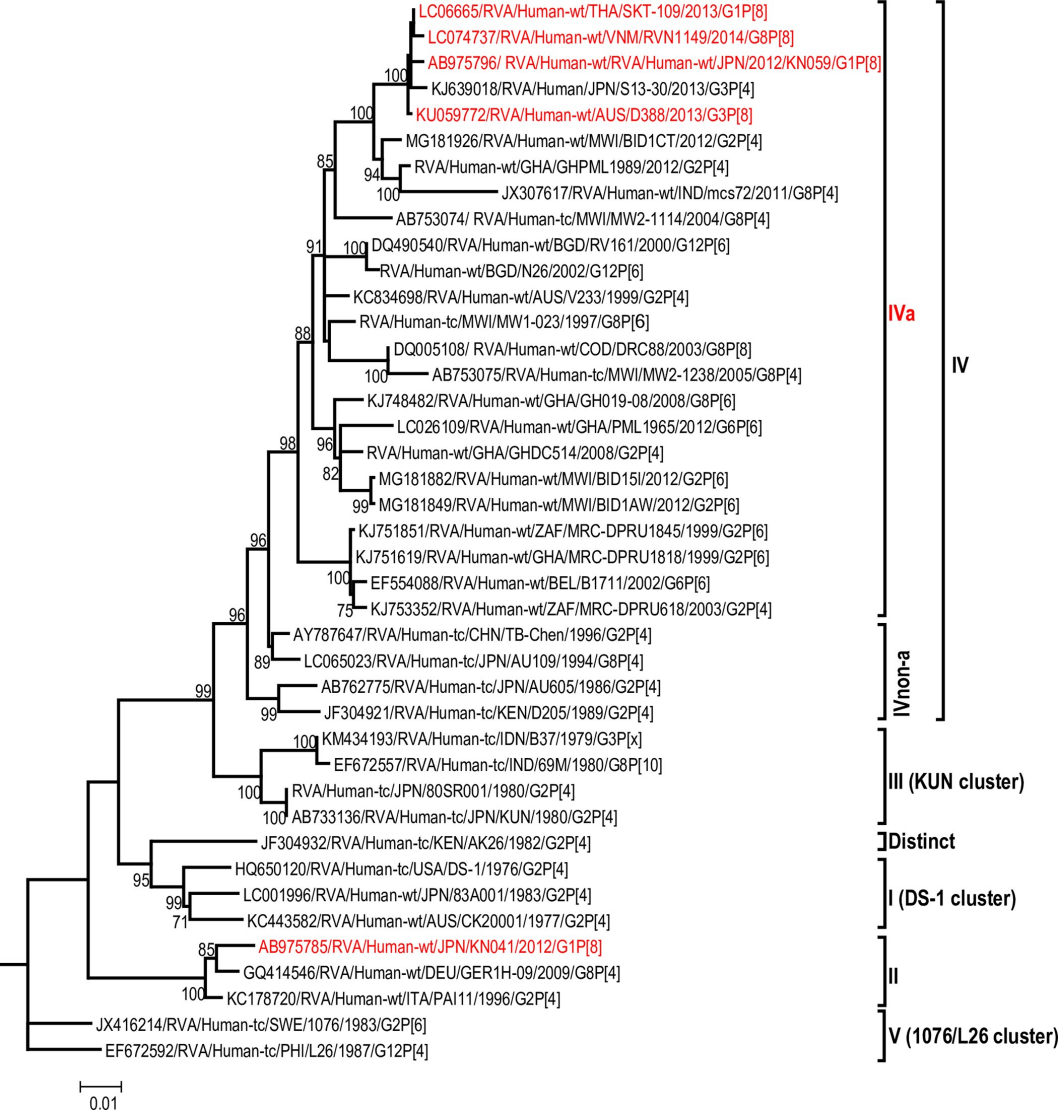
Reference phylogenetic lineage designation for the NSP1-A2 genotype of RVA strains. Phylogenetic tree was constructed using the maximum likelihood method with 1000 bootstrap replicate trials. The tree was rooted using the NSP1-A1 gene of the genotype 1 prototype strain Wa. Representative unusual emerging reassortant strains whose lineage constellations were determined in this study using the newly established phylogenetic framework are indicated in red font. Scale bar at the bottom of the tree represents genetic distance expressed as nucleotide substitutions/site.

**Fig 8:**
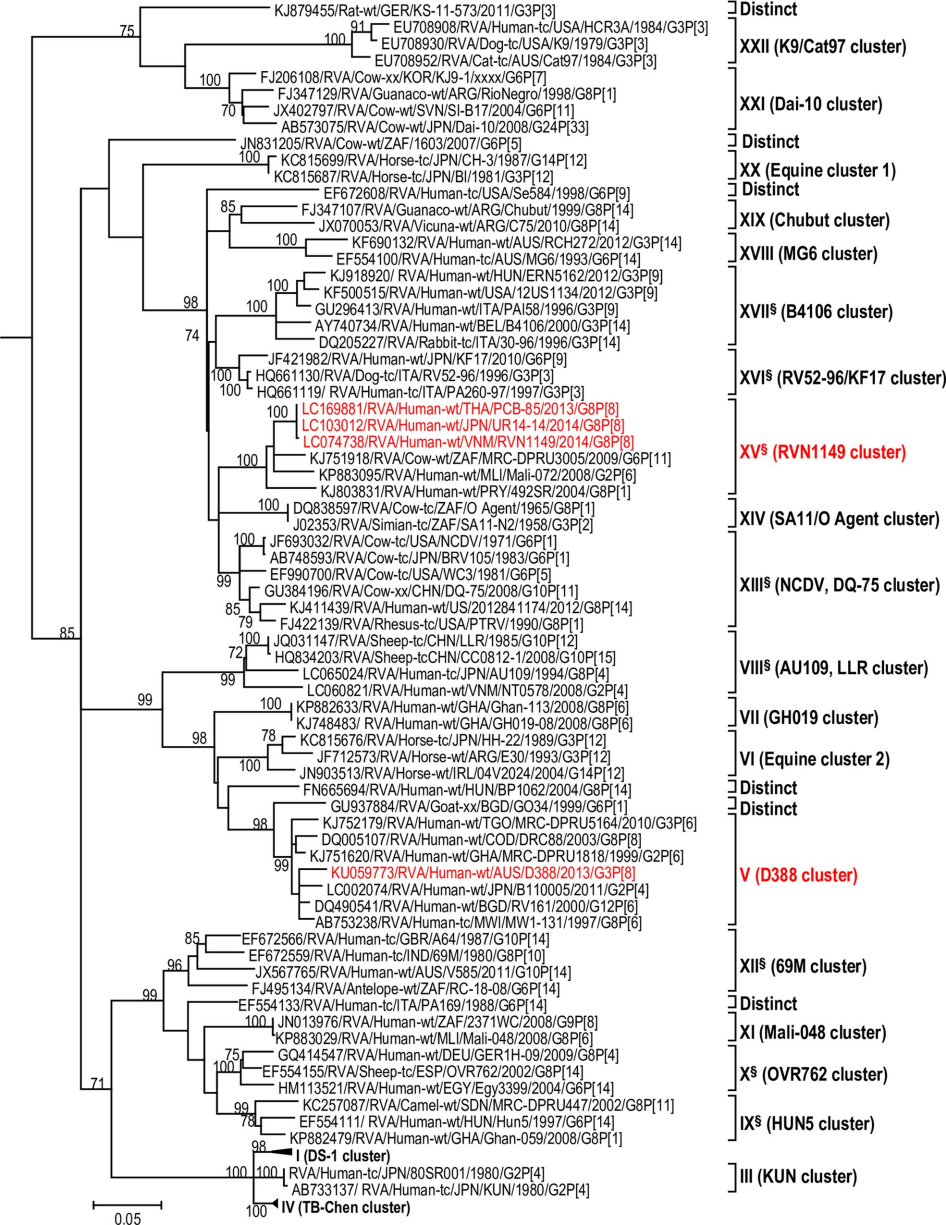
Reference phylogenetic lineage designation for the NSP2-N2 genotype of RVA strains of human and animal host species origin. Phylogenetic tree was constructed using the maximum likelihood method with 1000 bootstrap replicate trials. The tree was rooted using the NSP2-N1 gene of the genotype 1 prototype strain Wa. “Hybrid lineages” containing a mixture of human and animal RVA sequences are indicated with § in addition to the lineage number. Representative unusual emerging reassortant strains whose lineage constellations were determined in this study using the newly established phylogenetic framework are indicated in red font. Scale bar at the bottom of the tree represents genetic distance expressed as nucleotide substitutions/site.

**Fig 9:**
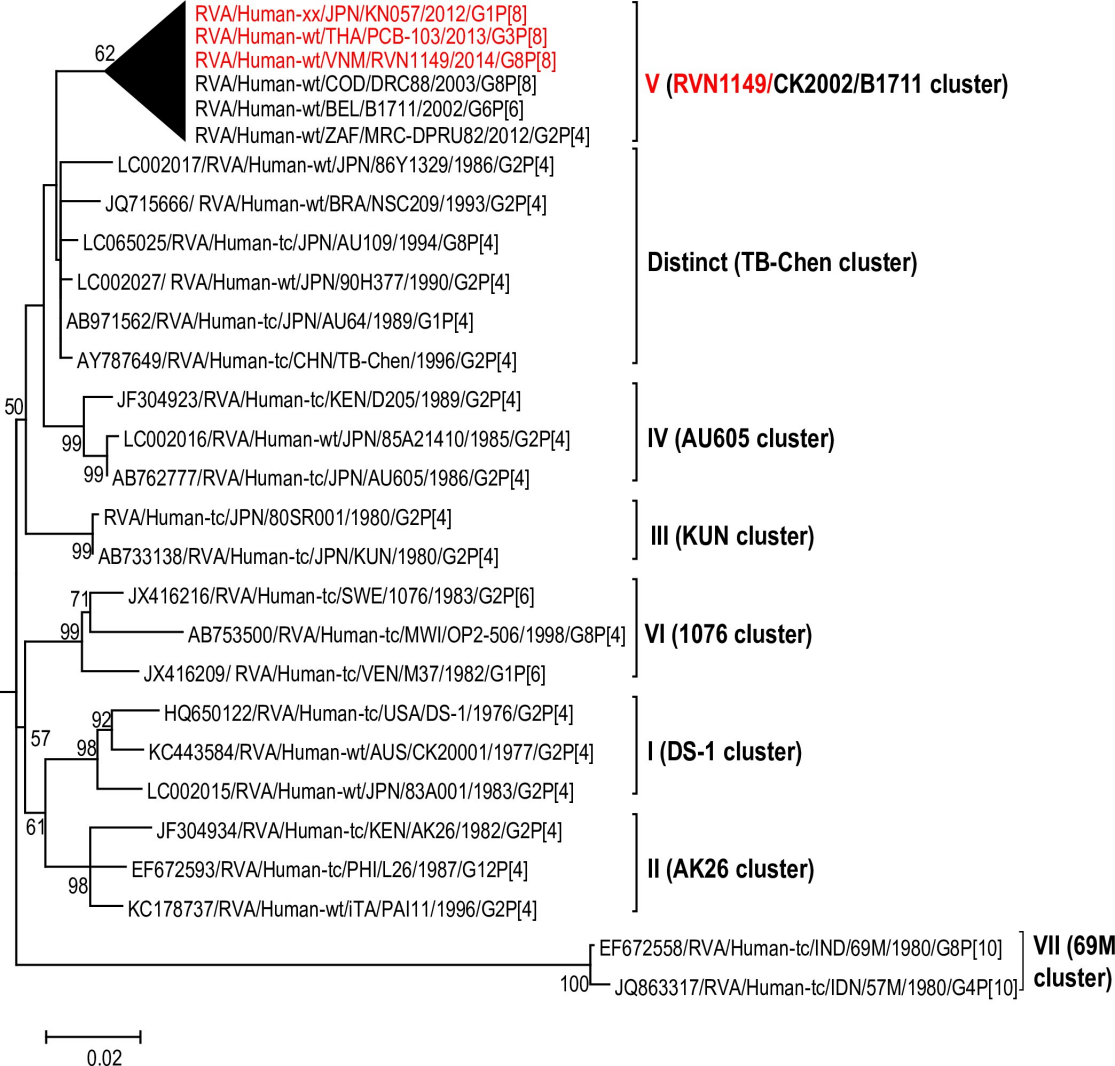
Reference phylogenetic lineage designation for the NSP3-T2 genotype of RVA strains. Phylogenetic tree was constructed using the maximum likelihood method with 1000 bootstrap replicate trials. The tree was rooted using the NSP3-T1 gene of the genotype 1 prototype strain Wa. Representative unusual emerging reassortant strains whose lineage constellations were determined in this study using the newly established phylogenetic framework are indicated in red font. Scale bar at the bottom of the tree represents genetic distance expressed as nucleotide substitutions/site.

**Fig 10:**
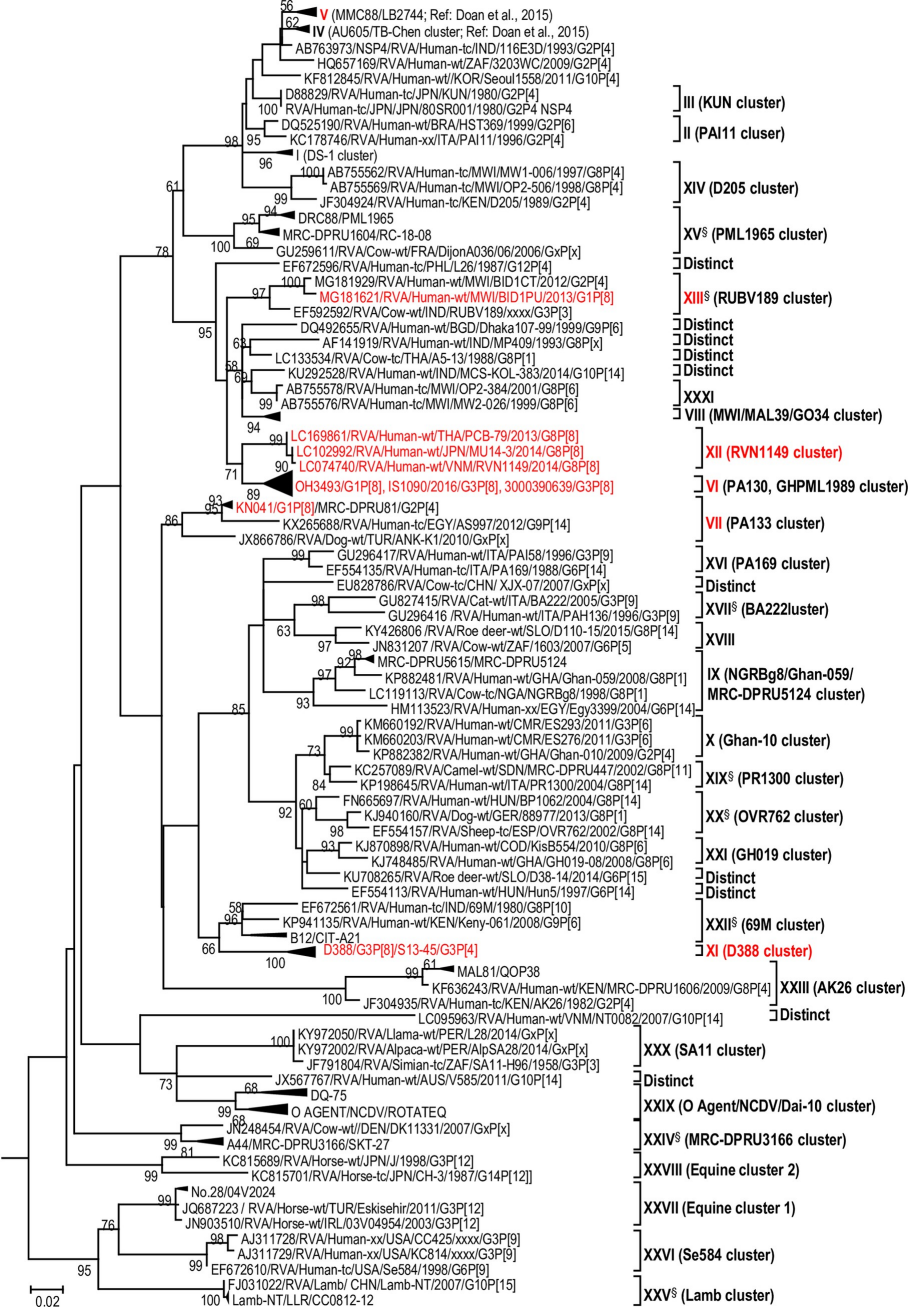
Reference phylogenetic lineage designation for the NSP4-E2 genotype of RVA strains of human and animal host species origin. Phylogenetic tree was constructed using the maximum likelihood method with 1000 bootstrap replicate trials. The tree was rooted using the NSP4-A1 gene of the genotype 1 prototype strain Wa. “Hybrid lineages” containing a mixture of human and animal RVA sequences are indicated with § in addition to the lineage number. Representative unusual emerging reassortant strains whose lineage constellations were determined in this study using the newly established phylogenetic framework are indicated in red font. Scale bar at the bottom of the tree represents genetic distance expressed as nucleotide substitutions/site.

**Fig 11:**
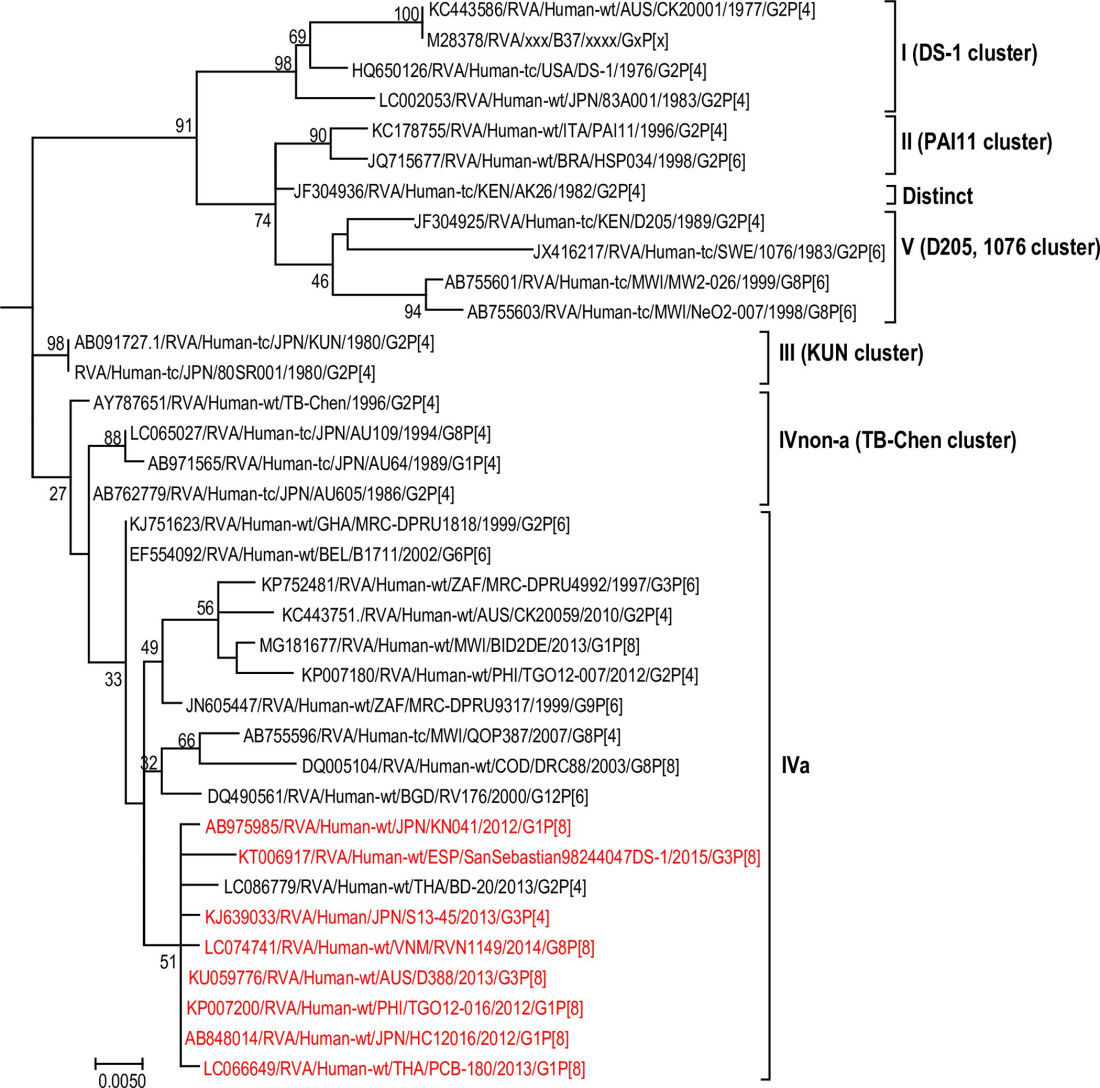
Reference phylogenetic lineage designation for the NSP5-H2 genotype of RVA strains. Phylogenetic tree was constructed using the maximum likelihood method with 1000 bootstrap replicate trials. The tree was rooted using the NSP5-A3 gene of the genotype 3 prototype strain AU-1. Representative unusual emerging reassortant strains whose lineage constellations were determined in this study using the newly established phylogenetic framework are indicated in red font. Scale bar at the bottom of the tree represents genetic distance expressed as nucleotide substitutions/site.

The maximum likelihood phylogenetic trees representing the framework for the genotype 2 backbone genes are shown in Figs 3–11. The number of lineages identified in the phylogenetic framework for each of the genotype 2 backbone genes was variable, ranging from five for the H2-NSP5 and the A2-NSP1 genes to 31 for the E2-NSP4 gene (Table 1). The nucleotide sequence diversity within a lineage ranged from 0.0 to 8.5%, whereas the diversity between lineages ranged from 1.6 to 18.3% (Table 1). The bootstrap support at the node was 70% or more in all lineages of four genotypes (i.e., R2, C2, A2, and N2) (Figs 3, 4, 7 and 8).

A few exceptions need to be noted in which bootstrap support was lower than 70%, yet lineages previously identified by Doan et al. [39] were maintained. This decision was necessary to avoid challenges with re-designation of lineages by previous authors [17, 24, 40–42] who have already applied Doan et al.’s scheme in assigning lineages to genotype 2 strains. In the I2 genotype (Fig 6), at the node of the three lineages, namely, lineages VIII, IX, XI, the bootstrap support was 34%, 68%, and 53% (values not shown since they are <70%), respectively. Although the bootstrap support for lineage VIII was very low, here came the sequences of RVA of probable artiodactyl origin with a nucleotide sequence divergence of 4% or less; thereby placing them in a group. In the M2 genotype (Fig 5), the support was 64% at the node of lineage VIII. In the T2 genotype (Fig 9), lineage V was noted to be supported at 62%, which was supported at >70% before the decimation and at 99% when only G2P[4] sequences were included for analysis (unrooted tree) [39]. In the E2 genotype (Fig 10), lineage V had a lower bootstrap value of 56% than the value (76%) when only G2P[4] sequences were included [39]. In the H2 genotype (Fig 11), lineage designation with statistically significant bootstrap supports was always difficult to achieve and it was kept in accordance with Doan, et al.’s designation.

As to the number of lineages, it was noted that lineages were more diverse in genes whose genotype was shared between human and animal RVA strains (i.e. genotypes R2, C2, M2, I2, N2, E2 corresponding to Figs 3–6, 8 and 10). In such phylogenetic trees, apart from clearly identified lineages that contained solely human or animal RVA sequences, there were also “hybrid lineages” (indicated with “§” in addition to the lineage number) that contained a mixture of both, indicating that interspecies transmission had occurred within those lineages. This phenomenon was common among the E2-NSP4 and N2-NSP2 genotypes. While phylogenetic analysis of the A2-NSP1, T2-NSP3 and H2-NSP5 genotypes generated five to seven lineages (Figs 7, 9 and 11; Table 1), that of R2-VP1, C2-VP2, M2-VP3, I2-VP6 generated 14 to 16, and that of N2-NSP2 and E2-NSP4 contained 22 and 31 lineages, respectively (Figs 3–6, 8 and 10; Table 1).

In addition, there were single sequences that were independent of the lineages. These sequences did not have closely related sequences in the GenBank hence were labelled “distinct” as they may represent lineages that are from RVA from less explored host species, lineages emerging, or lineages that have gone extinct. The E2-NSP4 tree exhibited the highest number of distinct taxa, followed by N2-NSP2, R2-VP1 and M2-VP3, I2-VP6 and C2-VP2 (Figs 3–11).

### Lineage constellations of unusual genotype 2 reassortant strains

The phylogenetic framework proposed in this study was used to describe the lineage constellations of the recently emerging unusual reassortant strains bearing the genotype 2 backbone genes such as G1P[8], equine-like G3P[8], equine-like G3P[4], bovine-like G8P[8], and G9P[4] strains (shown in red font in the phylogenetic trees; Fig 1). Fig 1 also contains the lineage constellations of representatives of both historical and contemporary G2P[4] strains previously published by Doan et al. [39], Do et al. [41], and Agbemabiese et al. [40], together with two G2P[4] strains recently reported by Jere et al. [13] to which lineages were assigned according to the current scheme. A few observations were made.

Firstly, using the currently updated phylogenetic framework, there were a total of 12 lineage constellations (considering the backbone genes) among 27 emergent reassortant strains (fully representing all the emergent strains). There were four constellations in G1P[8] reassortant strains, three in G3P[8]/P[4] reassortant strains, two in G8P[8] reassortant strains, and four in G9P[4] reassortant strains. Only some G1P[8] and some G3P[8] reassortant strains shared completely the same lineage constellation, suggesting a common evolutionary history between these reassortant strains. On the other hand, G8P[8] and G9P[4] reassortant strains underwent different pathways from each other as well as from those of G1P[8] and G3P[8] reassortant strains.

Secondly, no G2P[4] strains detected before 2000 shared an identical lineage constellation with any of the emergent reassortant strains (Fig 1). There were five contemporary G2P[4] strains whose backbone lineage constellations were distinct from each other, yet any one of these five distinct lineage constellations was shared with at least one emergent reassortant strain (Fig 1). This observation suggests that all emergent reassortant strains were generated through reassortment with contemporary G2P[4] strains in the recent past after 2000.

Thirdly, unlike other emergent reassortant strains, there was no corresponding G2P[4] strain whose backbone lineage constellation was the same as G8P[8] reassortant strains. This was due to the absence of lineage VII in genotype I2, XI in R2, XV in N2, and XII in E2 of any of the contemporary G2P[4] strains (Fig 1).

## Discussion

Examining clinical and surveillance specimens of RVA infecting humans at the level of the whole genotype constellation has provided a grand view of Wa-like, genotype 1 RVA originating from porcine RVA and DS-1-like, genotype 2 RVA originating from bovine RVA in the evolutionary perspective [2]. Likewise examining human RVA at the level of lineage constellation should provide better insight into the evolutionary history of human RVA bearing genotype 2 backbone genes. Thus, this study proposes a phylogenetic framework with a set of reference sequences under which a given genotype 2 backbone gene can be classified at the lineage level.

By examining G2P[4] strains detected in Italy over a 26 year period at the lineage level across the whole genome, Giammanco et al. [38] revealed that the G2P[4] strains detected between 2004 and 2011 had a novel lineage constellation distinctly different from the one before 2000. They also suggested that contemporary G2P[4] strains possessed either single or multiple genome segments (VP1, VP3 and/or NSP4) likely derived from ruminant viruses through intra-genotype reassortment. Independently, Doan et al. [39] defined lineages based primarily on the topology of the lineages across the genes based on the 150 G2P[4] strains for which full genome sequences were then available in the GenBank database. They proposed a stepwise evolutionary hypothesis in which global G2P[4] strains underwent successive replacement of the predominant strains from lineage I to lineage IVa/ V in all genome segments with some strains after 2004 undergoing intragenotype reassortment in the VP3 and NSP4 genes. The lineage framework proposed by Doan et al. [39] was subsequently used to study the lineage constellations of the G2P[4] strains in Vietnam and Ghana where new lineages were linked to animal RVA origin [40, 41].

Thus, the creation of the lineage framework proposed in this study started by including all genotype 2 sequences of both human and animal RVA origin. This enabled assignment of otherwise unclassifiable sequences to the defined lineages, simplifying the description as well as comparison of phylogenetic relations of the sequences of interest. To take a few examples from the previous studies, the NSP2 gene of a Japanese G8P[4] strain with the genotype 2 backbone, the AU109 strain, was described as clustering into a lineage with Chinese lamb and goat RVA strains with a nucleotide sequence distance of 4.5% [50]. This sequence is described simply as belonging to N2-lineage VIII according to the proposed scheme. Similarly, Do et al. [41] described the NSP2 sequence of two G2P[4] strains detected in Vietnam as not belonging to any previously described lineage, and explored its origin by running the BLAST analysis to find that they were close to Chinese lamb strains with a nucleotide sequence distance of 4.9%. These Vietnamese NSP2 sequences are described simply as N2-lineage VIII, making the comparison with Japanese AU109 straightforward without lengthy description.

On the other hand, recently emerging G8P[8] reassortant strains with the genotype 2 backbone from Vietnam, Thailand, and Japan shared the same NSP2 gene distinct from any of previously known NSP2 sequences that were reported to have originated from bovine or donkey RVA strains [27]. Despite the fact that the Japanese G8P[4] strain, AU109 [50] and the Japanese G8P[8] strain, MU14-3 [25] shared the same genotypes except the VP4 gene, and that both NSP2 genes were unusual and of probable animal RVA origin, the NSP2 genes of AU109 and MU14-3 are described as belonging to NSP2-lineage VIII and NSP2-lineage XV (Figs 1 and 8), respectively according to the proposed scheme, thereby making their distinctness clear at the lineage level.

As to the numbering of the lineages, we kept the scheme proposed by Doan et al. [39] because it has already been used in several studies [11, 17, 40–42] despite the observation that some of the lineages I, II, III and IV are better grouped together depending on the gene due to small nucleotide sequence divergence.

The application of the lineage framework on the emergent G1P[8] double-gene reassortant strains bearing the genotype 2 backbone led to an interesting observation: when comparing the lineage constellations of the backbone genes of these unusual reassortant strains with those of contemporary G2P[4] strains, there was always at least one G2P[4] strain that had an identical lineage constellation in the backbone gene to that of the G1P[8] double-gene reassortant strain of four different lineage constellations (Fig 1). A hypothesis may follow that the double gene reassortants could also be generated through single reassortment events between a co-circulating, standard G1P[8] strain and G2P[4] strain; alternatively, the double gene reassortants could have been generated through reassortment events occurring in succession with co-circulating G2P[4] strains. In this regard it is worthy of mentioning that Fujii et al. [10] encountered one G1P[8] double-gene reassortant strain detected in Japan (strain KN041 in Fig 1) in which the NSP1 and NSP4 genes were distinctly different from the rest of apparently monoclonal, 48 G1P[8] double-gene reassortant strains (one representative of which is KN039 in Fig 1). They interpreted that KN041 had resulted from further ressortment events between a KN039-like double-gene reassortant and a locally-circulating, genotype 2-bearing strain. However, an alternative possibility may be that KN041 was generated through a single reassortment event independent of the reassortment event that generated KN039-like strains. The failure to notice the alternative possibility comes from their inability to find a G2P[4] strain that had the same lineage constellation because they did not characterise KN041 at the lineage level due to the lack of a reference framework.

Concerning the host species origin of the sequences within each of the genotype, it was noted that three genes, namely the A2-NSP1, T2-NSP3 and H2-NSP5 genes, which all code for non-structural proteins, were limited to human RVA strains. These genes have much smaller number of lineages than the genes carried by RVA of diverse host species origin. In addition, between-lineage and within-lineage diversities of these three genes were small.

Even though the primary purpose of the lineage framework is not to speculate the host species origin of a lineage, some suggestions can still be made. For example, the topologies of the VP1, VP6, NSP2, and NSP4 trees suggest that (i) some of the new lineages emerged from circulating genotype 2 strains of animal host species origin; e.g., NSP2 lineage XV, and NSP4 lineage XIII (ii) the true host species origins of some lineages were inconclusive even though they share common ancestors with animal or animal-like human RVA sequences in the distant past; e.g., VP6 lineage VII, (iii) the origin of some of these new lineages was not clear due to the limited sequence data available; e.g., VP1 lineage XI compared to the G2P[4] strain PAI11/1996.

## Conclusion

This study proposes a refined phylogenetic framework for lineage designation for the genotype 2 backbone genes. Applying this new lineage framework to the analysis of emerging, unusual G1P[8], G3P[8] and G8P[8] and G9P[4] reassortant strains revealed that they share lineage constellations with contemporary G2P[4] strains, lending a strong support to the hypothesis that such unusual genotype 2 strains originated primarily from ressortment events in the recent past involving contemporary G2P[4] strains as one parent and ordinary genotype 1 strains or animal RVA strains as another. The lineage framework with selected reference sequences will help researchers to identify the lineage to which a given genotype 2 strain belongs, and track the evolutionary history of common and unusual genotype 2 strains in circulation.

## Supporting information

S1 Fig Host distribution of genotype 2 genes retrieved from the GenBank using the virus variation rotavirus resource. Sequences were retrieved using the virus variation resource based on the search criteria described in the results. Frequency of occurrence of each host species within each genotype 2 gene was tallied and plotted against the genome segments. The legend on the right side indicates the Latin names of the host species together with the corresponding colour in the histogram.
(PPTX)

S1 File Reference strains for phylogenetic framework for genotype 2 genes. (ZIP)
