# Supplementary material for: Sub-genotype phylogeny of the non-G, non-P genes of genotype 2 Rotavirus A strains
Source: PLoS One. 2019 May 31;14(5):e0217422. doi: 10.1371/journal.pone.0217422 (PMC6544246; doi:10.1371/journal.pone.0217422)
Supplement: S1 Fig — Sequences were retrieved using the virus variation resource based on the search criteria described in the results. Frequency of occurrence of each host species within each genotype 2 gene was tallied and plotted against the genome segments. The legend on the right side indicates the Latin names of the host species together with the corresponding colour in the histogram. (PPTX) [file pone.0217422.s001.pptx]

## Slide 1
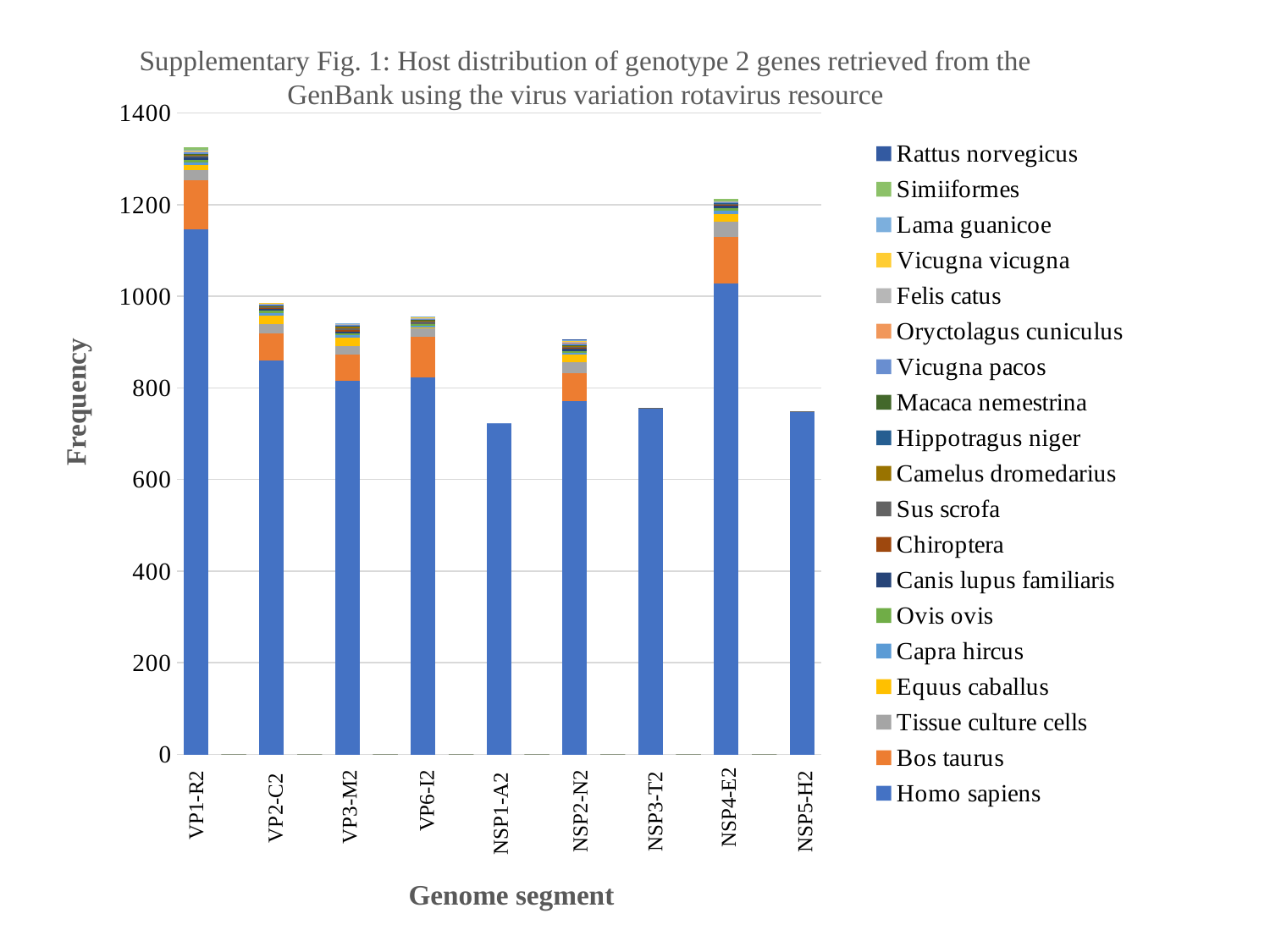

Supplementary Fig. 1: Host distribution of genotype 2 genes retrieved from the GenBank using the virus variation rotavirus resource
### Chart
| Category | Homo sapiens | Bos taurus | Tissue culture cells | Equus caballus | Capra hircus | Ovis ovis | Canis lupus familiaris | Chiroptera | Sus scrofa | Camelus dromedarius | Hippotragus niger | Macaca nemestrina | Vicugna pacos | Oryctolagus cuniculus | Felis catus | Vicugna vicugna | Lama guanicoe | Simiiformes | Rattus norvegicus |
|---|---|---|---|---|---|---|---|---|---|---|---|---|---|---|---|---|---|---|---|
| VP6 | 1146.0 | 107.0 | 22.0 | 11.0 | 7.0 | 4.0 | 6.0 | 0.0 | 5.0 | 1.0 | 1.0 | 1.0 | 3.0 | 2.0 | 1.0 | 1.0 | 2.0 | 6.0 | 0.0 |
| | 0.0 | 0.0 | 0.0 | 0.0 | 0.0 | 0.0 | 0.0 | 0.0 | 0.0 | 0.0 | 0.0 | 0.0 | 0.0 | 0.0 | 0.0 | 0.0 | 0.0 | 0.0 | 0.0 |
| VP1 | 860.0 | 59.0 | 21.0 | 17.0 | 7.0 | 5.0 | 4.0 | 2.0 | 2.0 | 2.0 | 1.0 | 1.0 | 1.0 | 1.0 | 1.0 | 1.0 | 0.0 | 0.0 | 0.0 |
| | 0.0 | 0.0 | 0.0 | 0.0 | 0.0 | 0.0 | 0.0 | 0.0 | 0.0 | 0.0 | 0.0 | 0.0 | 0.0 | 0.0 | 0.0 | 0.0 | 0.0 | 0.0 | 0.0 |
| VP2 | 816.0 | 57.0 | 19.0 | 17.0 | 6.0 | 4.0 | 4.0 | 4.0 | 4.0 | 2.0 | 2.0 | 2.0 | 1.0 | 1.0 | 1.0 | 1.0 | 1.0 | 0.0 | 0.0 |
| | 0.0 | 0.0 | 0.0 | 0.0 | 0.0 | 0.0 | 0.0 | 0.0 | 0.0 | 0.0 | 0.0 | 0.0 | 0.0 | 0.0 | 0.0 | 0.0 | 0.0 | 0.0 | 0.0 |
| VP3 | 823.0 | 89.0 | 19.0 | 1.0 | 4.0 | 4.0 | 0.0 | 0.0 | 6.0 | 1.0 | 1.0 | 1.0 | 2.0 | 1.0 | 1.0 | 1.0 | 2.0 | 0.0 | 0.0 |
| | 0.0 | 0.0 | 0.0 | 0.0 | 0.0 | 0.0 | 0.0 | 0.0 | 0.0 | 0.0 | 0.0 | 0.0 | 0.0 | 0.0 | 0.0 | 0.0 | 0.0 | 0.0 | 0.0 |
| NSP1 | 723.0 | 0.0 | 0.0 | 0.0 | 0.0 | 0.0 | 0.0 | 0.0 | 0.0 | 0.0 | 0.0 | 0.0 | 1.0 | 0.0 | 0.0 | 0.0 | 0.0 | 0.0 | 0.0 |
| | 0.0 | 0.0 | 0.0 | 0.0 | 0.0 | 0.0 | 0.0 | 0.0 | 0.0 | 0.0 | 0.0 | 0.0 | 0.0 | 0.0 | 0.0 | 0.0 | 0.0 | 0.0 | 0.0 |
| NSP2 | 771.0 | 61.0 | 25.0 | 15.0 | 4.0 | 4.0 | 6.0 | 1.0 | 4.0 | 1.0 | 1.0 | 1.0 | 2.0 | 2.0 | 3.0 | 1.0 | 2.0 | 2.0 | 1.0 |
| | 0.0 | 0.0 | 0.0 | 0.0 | 0.0 | 0.0 | 0.0 | 0.0 | 0.0 | 0.0 | 0.0 | 0.0 | 0.0 | 0.0 | 0.0 | 0.0 | 0.0 | 0.0 | 0.0 |
| NSP3 | 755.0 | 0.0 | 0.0 | 0.0 | 0.0 | 0.0 | 0.0 | 0.0 | 1.0 | 0.0 | 0.0 | 0.0 | 0.0 | 0.0 | 0.0 | 0.0 | 0.0 | 0.0 | 0.0 |
| | 0.0 | 0.0 | 0.0 | 0.0 | 0.0 | 0.0 | 0.0 | 0.0 | 0.0 | 0.0 | 0.0 | 0.0 | 0.0 | 0.0 | 0.0 | 0.0 | 0.0 | 0.0 | 0.0 |
| NSP4 | 1028.0 | 101.0 | 33.0 | 17.0 | 7.0 | 6.0 | 6.0 | 2.0 | 2.0 | 1.0 | 1.0 | 1.0 | 1.0 | 1.0 | 1.0 | 0.0 | 0.0 | 5.0 | 0.0 |
| | 0.0 | 0.0 | 0.0 | 0.0 | 0.0 | 0.0 | 0.0 | 0.0 | 0.0 | 0.0 | 0.0 | 0.0 | 0.0 | 0.0 | 0.0 | 0.0 | 0.0 | 0.0 | 0.0 |
| NSP5 | 749.0 | 0.0 | 0.0 | 0.0 | 0.0 | 0.0 | 0.0 | 0.0 | 1.0 | 0.0 | 0.0 | 0.0 | 0.0 | 0.0 | 0.0 | 0.0 | 0.0 | 0.0 | 0.0 |NSP3-T2
VP3-M2
VP6-I2
NSP4-E2
VP1-R2
NSP2-N2
NSP5-H2
VP2-C2
NSP1-A2
Frequency
Genome segment
